# Practising proportionate universalism – a study protocol of an extended postnatal home visiting programme in a disadvantaged area in Stockholm, Sweden

**DOI:** 10.1186/s12913-017-2038-1

**Published:** 2017-01-28

**Authors:** Bo Burström, Anneli Marttila, Asli Kulane, Lene Lindberg, Kristina Burström

**Affiliations:** 1 Department of Public Health Sciences, Equity and Health Policy Research Group, Karolinska Institutet, Stockholm, SE 171 77 Sweden; 2 Stockholm County Council Health Services, Stockholm, Sweden; 3 Department of Learning, Informatics, Management and Ethics, Health Outcomes and Economic Evaluation Research Group, Karolinska Institutet, Stockholm, Sweden

**Keywords:** Child health services, Disadvantaged areas, Equity, Extended home visiting, Health care need, Inequalities, Social services

## Abstract

**Background:**

In spite of a well-developed welfare system in Sweden, there are important health divides between residential areas in Stockholm county, with shorter life expectancy in disadvantaged areas. These socioeconomic and health divides also affect children. Extra efforts and organized collaboration by different authorities are required to meet the greater needs of children growing up in these areas.

**Methods/design:**

This article reports on the programme logic and evaluation design of an extended postnatal home visiting programme in collaboration between child health services and social services in the Rinkeby area, Stockholm, Sweden, where a large proportion are recent immigrants and more than 50% are at-risk of poverty. The intervention consists of five extra home visits when the child is aged between 2–15 months, jointly by a child health nurse and a social service parental advisor, offered to all parents of first-born children attending Rinkeby child health centre. Parents of first-born children attending child health centres in neighboring areas serve as controls. The evaluation will use a mixed methods approach, including participant observation, in-depth interviews, interviews using structured questionnaires, review and analysis of child health records and records of health care utilization.

**Discussion:**

The intervention has so far been very positively received by the parents (95% participation rate), who seem to perceive that they actually benefit from participating, and also from staff in child health services and social services who find this approach to be in line with their professional intentions. The staff members interviewed also appreciate the inter-professional collaboration. The intervention has sparked activities also in other sectors (the local library, the open child day care centre) of the local area.

The timing of the intervention, at the start of the child’s life, may be well suited to support parents in reorienting themselves and finding a positive parenting role, to the benefit of the development of the child. The intervention may be seen as a concrete example of “proportionate universalism”, as a strategy to reduce inequalities in health – applying a universal intervention with increased intensity in groups that have a greater need for it.

**Trial registration:**

The study was retrospectively registered (11 August 2016) in the ISRCTN registry (ISRCTN11832097 DOI:10.1186/ISRCTN11832097).

## Background

Sweden has a well-developed welfare system, based on high-quality services that should be available for all. In recent decades however, there have been many market-oriented changes to the system and deviations from the principle of allocation of resources based on need [1]. Overall, health is improving in the population, with increasing longevity in most groups. Nonetheless, social inequalities and accompanying health inequalities prevail [2]. Inequalities in health tend to be particularly pronounced in metropolitan areas of the country. There are important health divides between residential areas in Stockholm county, with differences in life expectancy of 4 to 6 years between disadvantaged and more affluent areas [3]. These socioeconomic and health divides also affect children.

Children growing up in socially and economically disadvantaged areas tend to face more difficult circumstances than children in other areas. The city district of Rinkeby-Kista, Stockholm municipality, comprises some 45,000 people and consists of the areas Rinkeby, Kista, Akalla and Husby. A large proportion of the population are recent immigrants, a higher proportion report a limiting longstanding illness and a lower proportion of working age adults are gainfully employed (52% in Rinkeby-Kista compared to the county average 75%) [3]. Long-term social exclusion, difficulties in finding housing and very limited economic resources in the family may have detrimental effects on children.

About 95% of the children in Rinkeby have foreign background, and some 56% of children in Rinkeby live in households which have an income less than 60% of the median, or receive social assistance, compared to 12% on national level [4]. According to a recent child health report in Stockholm county, 60% of children aged less than 6 years in Rinkeby belonged to a household with a disposable income in the lowest quintile, which in turn was associated with other adverse outcomes, such as lower participation in parental support groups, higher exposure to tobacco smoke and higher prevalence of dental caries at 3 years of age. The proportion of children born 2010–2015 where child health services have reported children to social services, or collaborate with social services was 34 per 1000 in RInkeby, compared to the average of 6 per 1000 in Stockholm county [5]. According to statistics from social services, the most common cause (one third of reports) for reporting was domestic violence [6].

In disadvantaged areas, extra efforts are needed by different authorities to meet the greater needs of the population. This is also in line with the recommendations of “proportionate universalism”, in the Marmot review in the United Kingdom [7]. There is also need for organized collaboration between the different relevant local services (e.g. child and maternal health services and social services) in order to improve children’s health [6]. However, the reimbursement system for child health services in Stockholm County Council disregards the greater needs in disadvantaged areas and provides the same amount for children living in disadvantaged and in more affluent areas. Hence, although the burden of work for child health services is greater in disadvantaged areas, the available resources do not match the need. Some indicators are presented in Table 1, where data are shown for the city district of Rinkeby-Kista and the neighboring city district Hässelby-Vällingby and Stockholm County as a whole for comparison.

**Table 1:** Comparison of child health care needs and provision of staff in different areas

Area	Children/fulltime child health nurse	Newborns/fulltime child health nurse	Index of care burden^a^	Purchasing power index^b^
Rinkeby-Kista	374	60	2.0	2.9
Hässelby-Vällingby	427	72	1.3	1.4
Stockholm County	432	72	1.0	1.0

Source: Child health report 2013, Stockholm County Council

^a^High value – greater burden of care

^b^High value – low purchasing power

Young children in disadvantaged areas are in some aspects less healthy and make on average more visits to emergency wards than children in other areas. Differences in inpatient episodes are less systematic. Data are shown for the residential areas of Rinkeby and Hässelby-Gård (a neighboring area) and Stockholm County as a whole (Table 2).

**Table 2:** Average number of outpatient visits and average number of inpatient episodes per child <1 year of age and 1–2 years of age, by area (Source: own analyses of Stockholm County Council health care administrative data, 2013)

Area	Average visits/child <1 year	Average visits/child 1–2 years	Average inpatient episodes/child <1 year	Average inpatient episodes/child 1–2 years
Rinkeby	1.60	2.01	0.24	0.09
Hässelby Gård	1.29	1.17	0.21	0.09
Stockholm County	1.15	1.24	0.28	0.08

In Sweden health services are a responsibility of county councils and regions, whereas social services are the responsibility of municipalities. Since September 2013 the Stockholm county council child health services and Stockholm municipality local social services in Rinkeby collaborate in a project where Rinkeby child health center offers an intervention in the form of intensified postnatal home visiting to all first-born children of parents in the area from 1 September 2013–31 December 2014. The project initially had financial support by the Public Health Agency of Sweden, with the objective to improve conditions for good mental health and development among children growing up in Rinkeby, through an early offer of individual support to their parents. The project was also expected to have beneficial impact on parental health, parental self-efficacy and social participation of the parents.

The present article describes the background and rationale for the intervention and the evaluation design to assess the impact of the intervention.

## Methods/design

**Fig. 1:**
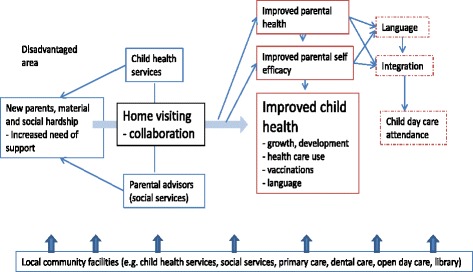
Programme logic model

Figure 1 depicts the rationale and programme logic of the intervention, which was the increased need for support among first-time parents in the Rinkeby area, many of who are recent immigrants and face material and social hardship, language and other barriers. Many also do not have gainful employment or an extensive social network.

For different reasons, the rate of participation in interventions is often lower in disadvantaged areas than in other areas [8]. However, many in the group of first-time parents have an increased need of support in their new role as parents and may therefore be assumed to be positive to participating in an intervention providing such support. Postnatal home visiting is evidence-based and has considerable support in the literature [9–12]. The intervention is expected to strengthen parents’ knowledge about children, improve the interaction between parents and children, increase the contacts of parents with other relevant societal actors, and strengthen their self-efficacy and well-being. Through this the health and well-being of children can also be improved [13].

Earlier studies of similar interventions [9–11] have had longer follow-up time, but noted improvements among both children and parents, in terms of mental health, reduced social assistance dependency, increased mastery among mothers and better interaction between mothers and children. In studies from the United States early positive effects have been noted among children regarding reduced emotional vulnerability at age 6 months, less language delays and increased mental development [11].

More recently this type of postnatal intervention was also shown in a randomized controlled trial in the United States in a population with high rate of poverty, to lower costly emergency care and improve family outcomes by infant’s age six months [12].

A recent review found that home visiting programmes may be particularly beneficial for socially high-risk families with young children [14]. Early initiation (even before birth), and increased number of visits improves development and health outcomes for certain groups of children. The review concluded that the dose of the intervention should be further studied, as well as the issue of retention of participants in the programme [13].

In Sweden, all parents are offered a child health programme of regular contact with child health services through planned clinic visits from the birth of the child until the child reaches six years of age. The programme includes one home visit about two weeks after birth, which is usually appreciated and accepted by most parents. Hence, there is a tradition for child health services to do home visiting. Building on this, the intervention assumed that additional home visiting during the first 15 months of the child’s life might be a beneficial way to support parents – allowing questions and opportunities for guidance and support for parents at different stages of the child’s development, advice on how, where and when to seek health care and on vaccinations. Although not a regular feature in the child health programme, it may be assumed that parental advisors from social services might provide additional beneficial advice and support regarding parenting, attachment, play and interaction with the child.

To our knowledge, no similar project has been carried out in Sweden. In the Rinkeby area the social services receive reports about social problems and children not faring well, at a higher rate than in other areas in Stockholm. However, social services sometimes come into the process only when the children are older, and would like to focus on early preventive interventions instead. Collaboration between child health services and social services in a postnatal home visiting programme could therefore be a beneficial addition to home visiting only by child health services, enabling social services to offer parental guidance and support to all parents in the target group, instead of only those who had difficulties.

In addition, the intervention links and makes use of other existing local community services and facilities in the area (e.g. child health services, social services, primary care, dental care, open day care facilities and the local library).

Although there is scientific evidence of the benefits of intensive home visiting by child health nurses [9–11], there is less experience and evidence of the benefits of such early home visiting by social services parental advisors. The project assumed that the combination of the two would benefit both the child’s health and growth, and parental self-efficacy and parental health. Positive effects on parental self-efficacy and health would then also have positive effects on child health and growth. In addition, the intervention might stimulate parents to learn Swedish language and contribute to their improved integration into Swedish society, and may make parents more positive to having their children in day care where the children can get further stimulation regarding for example Swedish language and social interaction with other children.

However, no scientific evaluations have been made of the impact of a collaborative effort between child health services and social services in intensified postnatal home visiting, on the health and development of children and on the health, parental self-efficacy and social participation of parents. It is this knowledge gap the project intends to bridge.

## The intervention

The study is registered in the ISRCTN registry (ISRCTN11832097 DOI 10.1186/ISRCTN11832097), where further details may be found. A preliminary report has been published in Swedish [14]. All first-born children to parents listed with the child health center in Rinkeby (the intervention area) are offered to take part in the intervention. The decision to include all and not just a selection of first-born children was based on the high prevalence of social problems in the area, and in order to avoid stigmatization of participating parents. Furthermore the area has worse results than other areas in several indicators of child health (vaccination rates, overweight, dental caries). In addition to the regular child health programme, participating parents are offered five extra home visits during the child’s first 15 months by a child health nurse and a parental advisor. The offer is entirely voluntary to the parents.

If they agree, the parents sign a consent form where they agree to take part in a questionnaire-based interview when the child is aged 2–3 months and a similar follow-up interview at child’s age 15–18 months. Parents also agree that the researchers may trace the child’s patient records during the study period, and until the child turns 6 years. Parents are free to cancel their participation at any time.

### The extra postnatal home visits

Participating parents in the intervention area are offered five extra home visits by a child health nurse and a parent counsellor social worker, from infant’s age about 2 months until age 15 months. The detailed content of the home visits is described elsewhere [14]. Different themes are discussed during the home visits (child safety, child feeding, child attachment/interaction, parenthood, social network, self-care). Visual graphic material and dolls are used to facilitate communication and many visits are assisted by a language interpreter. The home visits allow the child health nurse and the family counsellor social worker to strengthen positive interaction between parents and their child, to advise parents on alternative courses of action when needed and not least to establish a trusting relation with the parents. The emphasis is on health promoting aspects. The local public library in Rinkeby donates an infant picture book to each child, for parents to read to them. The public dental services provide a toothbrush and toothpaste in order to stimulate parents to brush the teeth of the infants. The child health nurse and the parental advisor also inform parents about local activities for parents with infants (e.g. open daycare activities; reading activities organized by the library for parents and infants) and about other relevant local public activities.

## Planned evaluation – ethical issues and methodological challenges

Before the start of the project it was decided not to have a randomized control trial design, but that the intervention should be offered to all first-time parents attending the child health centre in the intervention area, and the evaluation was designed as a comparison between participating parents and their first-born child in the Rinkeby area, and parents and their first-born child attending another child health centre in neighboring control areas (Husby and Hässelby Gård in Stockholm municipality) that are offered the regular child health programme, with no planned involvement of social services. The rationale for having a facility-based design with comparison between areas was that it was considered potentially stigmatizing and unethical to single out which parents should be offered the intervention and not and that the intervention also would affect the whole child health centre and possibly the residential area.

The evaluation has a mixed methods approach, combining qualitative and quantitative methods of data collection and analysis [15, 16].

### Ethical permission

Ethical permission for the study for the project duration was granted by the Regional Ethics Committee, Stockholm (Dnr 2013/877-31/1), and for an extended time for follow-up of medical records until the child reaches the age of six years (Dnr 2014/1773-32). Informed consent has been obtained from parents for participation in the study.

### Process evaluation

The process evaluation has mainly a qualitative approach, and aims to systematically reflect how the intervention is carried out and experienced by participating parents and staff, including facilitating factors and obstacles. An evaluator has been part of the project from the initial phase, interviewing participating staff and documenting the progress of the project. The evaluator also participates in regular meetings between staff of the child health services and parental advisors from social services.

In addition, the content of home visits is discussed in relation to the different themes which are raised during the visits (child safety, child feeding, child attachment/interaction, parenthood etc.). Each home visit is documented independently by the child health nurse and the parental advisor, respectively. The process evaluation will also include analysis of this material.

A subgroup of participating parents are interviewed during the intervention period, regarding their health and the health of their child; the themes covered during home visits and visits to the child health center; their thoughts on parenthood, family, work situation, economy, other social relations and their expectations on public authorities. After the intervention parents will be asked what they think the intervention has contributed to them, or what they would have wanted instead.

The qualitative material will be analysed for its content, using appropriate methods [17–19].

### Effect evaluation

The effect evaluation has a mixed methods approach [15, 16], collecting data on parents and their children, among those in the intervention area as well as in control areas. Comparison will be made of data collected before and after, between those receiving and not receiving the intervention. However, early on the evaluator realized that in this multicultural context the different underlying views and ideas of the parents must be considered also in relation to the structured questionnaire-based interviews. Therefore the evaluator has noted down parents’ comments to terms and questions in the questionnaire, what questions they ask and words they perceive as difficult to understand. The survey responses will be analysed both qualitatively and quantitatively, with a mixed methods approach.

#### Questionnaire-based interview

An interview questionnaire is administered to parents when the child is 2–3 months and 15–18 months of age. Data collected in the interview questionnaire includes information on country of birth, length of residence in Sweden, length of education, employment status, type of income. In addition, parents answer questions on their self-rated health and health-related quality of life using the EQ-5D instrument [20], their social network and social support; their perception of being a parent, using a modified version of the Parental Self Efficacy instrument [21–23]. Parents also rate their child’s health; sleep and feeding issues. The second interview also asks about participation in public playschool and other activities. Other comments are also invited from interviewees at the end of the interview, and during the course of the interview as described above. Most of the interview questionnaires are administered in a standardized manner by the evaluator, which reduces interviewer bias. Many interviews are done with language interpreters.

#### Analysis of medical records

The effect evaluation also includes data from the child’s medical records, concerning feeding, growth; visits to child health center, emergency room services and visits to other types of health care, and treatment received. Furthermore there is information on referrals to other types of care (specialist medical services; child psychiatry; social services). The extraction of data and analysis of medical records is done in collaboration with a child health nurse and a pediatrician.

Child health will be measured through growth monitoring, adherence to the child health programme, other health care use (visits to clinics or in-patient episodes), vaccination coverage rates, dental caries and the occurrence of deviations from normal child development (in terms of language and functional abilities) at different ages, obtained from the child health records.

### Methodological challenges

There are a number of ethical and methodological challenges in the evaluation. Many of the parents who participate in the intervention are in a vulnerable position. Some are recent immigrants, new to Swedish society; some are undocumented and not known by authorities; others are struggling to make ends meet under very difficult social and economic circumstances. This means that it is particularly important that ethical issues are considered. Parents come from some 30–40 different countries and a large proportion of the interviews are done through language interpreters. Even where language translation works well (and it does not work well in all instances), there are difficulties in the understanding of certain words and concepts, going beyond language barriers.

There is high mobility among the participants, some of whom move out of the area and even out of the country, which may make follow-up difficult. Previous studies to assess impact of intensified home visiting have had longer follow-up time [9–11], and the short period of follow-up in the current evaluation may mean that some effects are missed.

In view of these difficulties the evaluation has a mixed-methods approach [15, 16] and consists of both a process evaluation and an effect evaluation based on a structured questionnaire interview, analysis of medical records and in-depth interviews with participating parents and staff. In order to better understand how the intervention is being implemented and perceived by both the participating parents and the staff involved, it is important for the evaluation team to have a strong presence in the setting where the intervention takes place, through participatory observation.

## Planned studies

A number of studies are planned and on-going, to address research questions relevant to the intervention, with regard to:

1. How the intervention is accepted by parents?
2. Which factors may facilitate or obstruct the implementation of the intervention in a disadvantaged area?
3. What are the short term (at child’s age 18 months) effects on child health and development of the intervention?
4. What are the short term (at child’s age 18 months) effects of the intervention on parents, with regard to their health-related quality of life and their parental self-efficacy?
5. How does the intervention affect the adherence to the child health programme and the number and type of visits to health care services for children?

The intervention is on-going since September 2013. The second round of interviews with parents is being finalized during 2016. When data collection is completed, comparisons before and after can be made, of questionnaire data in the intervention and comparison area.

## Preliminary findings so far

Unlike many other interventions undertaken in disadvantaged areas which may have low participation rates, the intensified postnatal visiting programme has been received very positively by the parents so far. It was expected that not all would participate because many parents struggle in difficult socioeconomic situation and live in uncertain situation (for example as asylum seekers). However, out of the 119 families offered to participate, 11 families moved out before the interview and only 7 have declined participation. A total of 101 first interviews have been carried out in the intervention area. In the control areas, 92 of 122 included families (75%) participated in the first interview.

In the intervention area the vast majority of parents are born outside Sweden and many have come to Sweden quite recently, some 50% of the mothers interviewed have arrived 2010 or later. Most interviewed mothers in the intervention area were born in Somalia; the other interviewees come from about 30 different countries (for example Eritrea, Ethiopian, Iraq, Uzbekistan, and Turkey). Among the parents participating in the intervention, seven were born in Sweden. In the control areas the majority are also born outside Sweden, in some of the countries listed above but also in some other countries like Morocco, Libya, Palestine, Mongolia, and Latvia [13].

There is great variation in the amount of schooling of the mothers interviewed in the intervention area, from a few weeks of school to 14 years or more. Some have academic degrees, but most have less schooling. In the comparison area a greater proportion of mothers so far report that they have more schooling. More than half of the first interviews have been made using an interpreter in the intervention are, and about a third so far in the control areas. There is considerable mobility among the parents, moving within and between different residential areas, and some moving abroad, both among the parents in the intervention and control areas. In both areas a number of undocumented migrants are part of the study, as well as parents seeking asylum or with rejected applications for residence permit [13].

Preliminary analysis of findings suggests that the parents report large basic needs in the intervention area (many lack housing, income, social contacts) which have not been met. All interviewed are happy to have had a baby, and appreciate receiving information from child health services related to becoming a parent. Some parents have a great need to talk about their difficulties and experiences during the delivery. Some mothers also report problems with anxiety, depression, lack of social network and social support. As this is their first child, many parents feel insecurity in their role as parent, especially when the baby gets ill; it is difficult to know how to act and where to go in that kind of situations [13].

The preliminary impression is that parents, as well as the professionals engaged, appreciate the intervention, which provides a chance to build a relation with the child health nurse and the parent advisor social worker. The nurses and social workers engaged in the extended postnatal home visiting programme find it rewarding to collaborate in the work, to learn from each other’s skills and appreciate that extended home visits facilitates a good relation with parents, which also makes it possible to build trust and discuss more sensitive issues with the parents [13].

## Discussion

The described intervention may be seen as both novel and original, in its attempt to improve mental and physical health among children growing up in a disadvantaged area, through collaboration between child health services and social services by aiming to strengthen parents and their self-efficacy in their new role. It builds on evidence from previous studies [9–12], and the approach of home visiting may be particularly beneficial for socially disadvantaged groups and their children [14]. The timing of the intervention, at the start of the child’s life, may be well suited to support parents in reorienting themselves and finding a positive parenting role, to the benefit of the development of the child.

The intervention has so far been very positively received by the parents, who seem to perceive that they actually benefit from participating, and also from staff in child health services and social services who find this approach to be in line with their professional intentions. The staff members interviewed also appreciate to collaborate between their respective professions. This approach builds on and strengthens existing services in the local community and may have positive synergistic effects. The limited extra resources needed for the intervention seem to result in a considerable benefit to both parents and professionals. An additional unintended effect of the intervention is that three child health nurses working in the intervention area have taken up studies on master level, on topics related to the intervention. Such increased competence development may also further reinforce the intervention, and is of importance for retaining staff in areas with high turnover.

The intervention in the Rinkeby area may be seen as a concrete example of “proportionate universalism”, as a strategy to reduce inequalities in health – applying a universal intervention with increased intensity in groups that have a greater need for it. A currently ongoing issue is how to convince child health care policy makers to provide the limited extra resources needed to continue the intervention as part of the regular work. Policy makers in social services have already integrated the cost of parental advisors into their regular budget.

There are several methodological challenges in relation to the evaluation of the intervention, which need to be overcome in order to properly assess the impact of the intervention, for example the use of language interpreters in the interviews. Should the project prove to have the positive effects which the preliminary results indicate, it could be an important contribution to the knowledge on how to improve health and reduce health inequalities among children in disadvantaged circumstances, by supporting and empowering their parents. A secondary effect of the intervention might be to shift health care utilization of families with children to a more appropriate level. Documenting and learning from the experience of the current project will also facilitate dissemination of the knowledge gained and inform others attempting a wider application of the approach to other settings.

## Conclusions

The timing of the intervention, at the start of the child’s life, may be well suited to support parents in reorienting themselves and finding a positive parenting role, to the benefit of the development of the child. The intervention may be seen as a concrete example of “proportionate universalism”, as a strategy to reduce inequalities in health – applying a universal intervention with increased intensity in groups that have a greater need for it.
